# Methodology for the Construction of the Bicyclo[4.3.0]nonane Core

**DOI:** 10.3390/molecules21101358

**Published:** 2016-10-12

**Authors:** Nicholas A. Eddy, Pranjali Ichalkaranje

**Affiliations:** Department of Chemistry, University of Connecticut, Storrs, CT 06269 USA; pranjali.ichalkaranje@uconn.edu

**Keywords:** triterpenes, triterpenoids, Diels-Alder, metathesis, Michael additions

## Abstract

The bicyclo[4.3.0]nonane scaffold, commonly known as a hydrindane, is a common structural motif found in many terpenoid structures and one that remains a challenge for synthetic chemists to elaborate with appropriate regio- and stereo-selectivity. Over the course of the study of terpene natural products, the elaboration of the hydrindane structure has seen progress on the utilization of both old and newer methods to achieve the desired outcomes. This review seeks to serve as a general overview of these methods, and detail specific examples.

## 1. Introduction

Throughout the vast array of structures that polyterpenes and polyterpenoids can exist in, a common motif that appears regularly has been the bicyclo[4.3.0]nonane (also called hydrindane) scaffold [1]. Notably, the hydrindane moiety often contains a significant portion of the stereochemistry contained in the natural product; thus presenting a wealth of opportunity for method development and posing a synthetic challenge [2]. Over the past forty years, different strategies have been employed to showcase the utility of reaction sequences leading to the hydrindane nucleus, with most targeting the thermodynamically unfavorable *trans* isomer. This review will focus on the general classes of reactions used for the synthesis of hydrindane cores, as well as examples of strategies employed.

**Figure 1:**
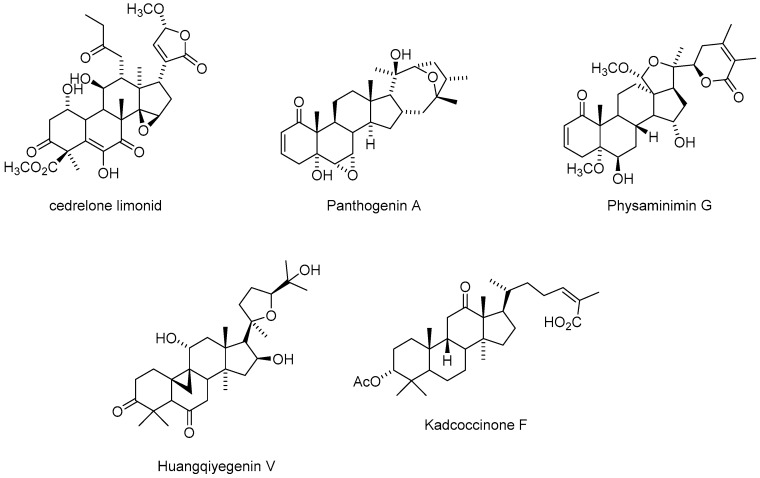
Examples of naturally occurring triterpenoids.

Natural products play a predominant role in medicine and nature, and often bear complex structural elements, e.g., fused ring systems, bridged structures, and contiguous stereocenters. Of particular interest is the hydrindane nucleus, which bears significant amounts of stereocenters in a broad range of bioactive natural products. Naturally occurring terpenoids, functionalized terpene derivatives, are not just a structural curiosity, but play a central role in medicinal chemistry [1,3]. Their biological activity can range from anti-tumor activity to effects on cardiovascular systems (cardiac glycosides) to anti-inflammation [4,5,6,7,8,9]. Due to these far ranging biological activities, the hydrindane scaffolds often become the target for development into drug-like molecules for the treatment of disease [10,11]. As the reader will note, the hydrindane scaffold is present throughout the examples shown in Figure 1. In light of the prevalence of this scaffold, it should be apparent that no small effort is made in the synthesis and utilization of this common structure across multiple targets.

The cedrelone limonids, isolated from *Trichilia P. Br. (Meliacae)*, possesses bioactivity against cancerous cell lines for leukemia (HL-60), hepatocellular carcinoma (SMMC-7721), lung cancer (A-549), breast cancer (MCF-7), and colon cancer (SW480) [9]. The isolated compounds from this study ranged in IC_50_ from 1 to 15.9 μM. Panthogenin A and B, isolated from *Dioscorea panthaica*, causes 100% insulin sensitization at 10 μg/mL [12]. Physaminimin acts an antibiotic and anti-inflammatory compound isolated from *Physalis minima* in southeastern China, and tested for nitric oxide synthase (NOS) activity in multiple cell lines [4]. Huangqiyegenin V also possess anti-inflammatory activity and has been tested for NOS inhibition [13]. Kadcoccinone F and its congeners have exhibited anticancer bioactivity against HL-60, SMMC-7221, A-549, MCF-7, and SW480 cell lines [14]. Ultimately, these are a small subset of the diverse scaffolds that triterpenes and triterpenoids may exist in, but serve as a representative set for the purpose of this review. Herein, this review will point to multiple methods of derivation and focus on the synthesis and use of the hydrindane system for biologically active, medicinally relevant, natural product synthesis.

## 2. Synthetic Strategies

### 2.1. Hajos–Parrish Dione

**Figure 2:**
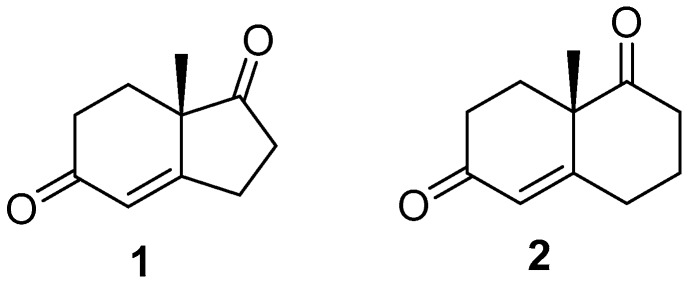
Structure of Hajos–Parrish dione (**1**) and Wieland–Miescher dione (**2**).

When considering the structures of the natural products shown in Figure 1, the reader should note that a large portion of the molecules’ stereochemistry is contained in the hydrindane scaffold, giving it a range of structural complexity that can be challenging to synthesize. This prompted the development of the Hajos–Parrish dione (**1**) and its 6,6-fused analog, the Wieland–Miescher diketone (**2**), shown in Figure 2. The present section will detail the development, use, and elaboration of scaffolds stemming from **1** for natural product synthesis.

**Scheme 1:**
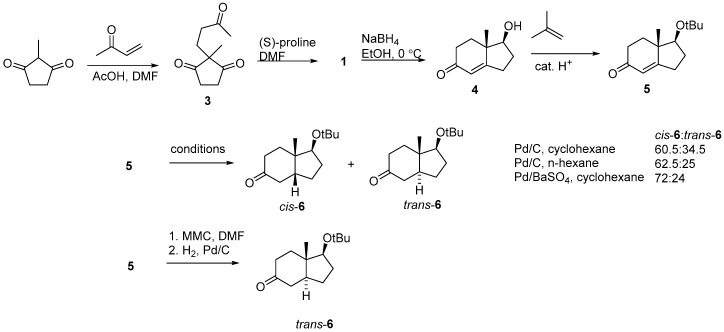
Synthesis and reduction of **1** to *trans*-hydrindane derivative **6**.

In 1974, Hajos and Parrish described the synthesis of **1** in an effort to enantioselectively synthesize the *trans*-hydrindane present in steroids (see Scheme 1) [15]. Here it was found that Michael addition of 2-methyl-1,3-cyclopentadione with but-3-ene-2-one afforded the intermediate **3**, which was subsequently transformed into **1** through the action of proline. A benefit to this sequence is that either enantiomer of **1** can be synthesized efficiently. Further treatment of **1** with NaBH_4_ in cold ethanol selectively reduced the more sterically free ketone to **4**, which was then protected as the *tert*-butyl ether **5** through acid catalyzed reaction with isobutylene [16]. Upon reduction of **5** with mild reduction conditions for palladium catalyzed hydrogenation, it was found that the *cis*-hydrindane (*cis*-**6**) was formed in preference to the *trans* isomer (*trans*-**6**). In consideration of **5**’s structure, the authors attributed the reaction preference to the more open β-face being presented over the α during the reduction [17]. Under the best conditions, only 34.5% of *trans*-**6** was found from palladium catalyzed reduction in cyclohexane. Derivatization of **5** with methyl magnesium carbonate (MMC), followed by the same reduction conditions was able to selectively produce *trans*-**6**, which upon treatment with 2 M HCl undergoes hydrolysis and decarboxylation to afford *trans*-**6**. The isolated *trans*-hydrindane was carried forward through a series of Michael additions and annulations to yield the desired 19-nor-steroid systems for study.

**Scheme 2:**
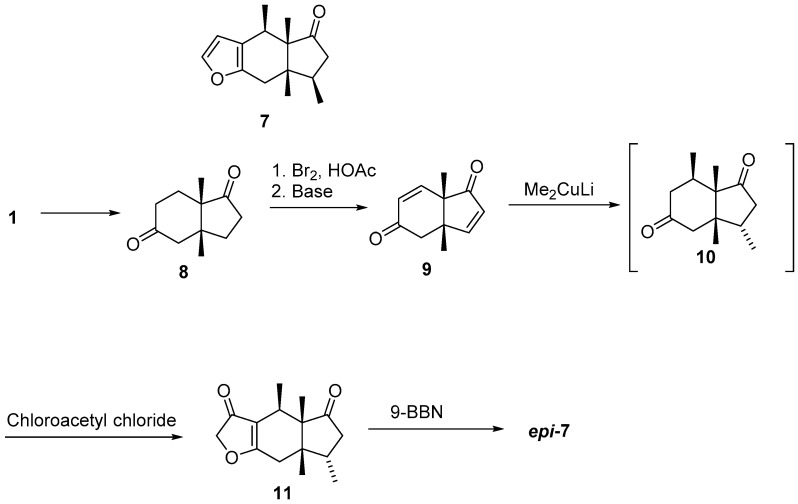
Synthesis of 7-*epi*-pinguisone (*epi*-**7**).

Other terpenoid systems benefit from **1**. 7-*epi*-Pinguisone (*epi*-**7**) exhibits insect anti-feedant activity and was synthesized in 1981 by Jommi (see Scheme 2) [18]. In this work, the authors relied upon the nature of the open β-face to selectively add a second methyl group in the *cis* configuration, giving **8**. Treatment of **8** with an excess of bromine in acetic acid, and further elimination provided **9**. Exposure to an excess of lithium dimethylcuprate allowed for **10** with four contiguous stereocenters formed with the appropriate stereochemistry. The authors attribute the high level of induction for **10** to stem from the Drieding model of **9**, where the steric hindrance of the α- and β-face of the molecule are similarly hindered, and thus the selectivity of the reaction is governed by the steric bulk of the cuprate. Compound **10**, isolated during exploratory reactions, was acylated through its enolate directly after cuprate addition, and spontaneously cyclized to form dione **11** without isolation of the intermediate dione. Reduction with 9-BBN on **11** afforded *epi*-**7**.

**Scheme 3:**
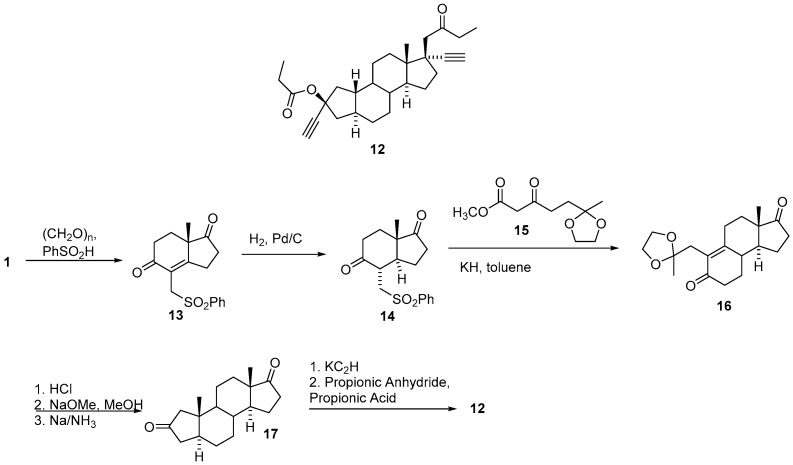
Synthesis of dinordin (**12**).

Unnatural A-19-nor-steroids provide another class of biologically active molecules. Anordrin, an androstane like molecule, has been used for contraception (see Scheme 3). Work done in 1983 by Crabbe on an analogous system, dinordrin (**12**), showcased further use of **1** in synthesis [19]. Beginning with **1**, the reaction was sulfonated to afford **13**, and then reduced to **14** with H_2_ and Pd/C. Condensation with **15**, formed by ethyl acetoacetate and ethylene glycol, afforded **16** after annelation. Subsequent deprotection and aldol condensation led to androstane core **17**. Ethynylation and trapping with propionic anhydride led to dinordrin **12**.

**Scheme 4:**
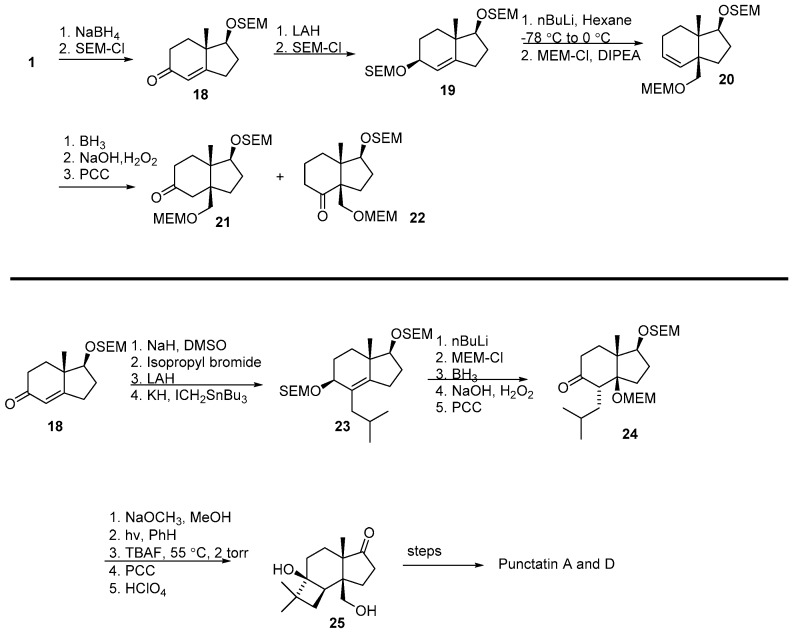
Synthesis of hydrindane scaffold for Punctatin A and D.

Paquette followed with a synthesis of punctatins A and D (see Scheme 4) [20]. Using **1**, reduction of the more hindered ketone followed by protection with tributyl chloromethylstannane (SEM-Cl) led to **18**. Treatment with LiAlH_4_, followed by protection of the resulting alcohol with SEM-Cl led to the distannyl **19**. Lithiation at −78 °C with warming allowed for Still’s [2,3]-Wittig rearrangement, which was protected as the methoxyethoxymethyl (MEM) ether (**20**). Hydroboration followed by oxidative cleavage leading to **21** found issues with regioselectivity from **1**, typically giving a mix with **22**. In an effort to increase the regioselectivity of the latter steps, **18** was derivatized with isobutyl bromide, giving **23**. Under the same sequence of reduction and protection as prior, the authors were able to generate **24**. Further elaboration through their conditions for Still’s [2,3]-Wittig rearrangement and hydroboration/oxidation sequence found better regioselectivity for the resulting ketone. Photolysis was attempted, and found to give the improper stereochemistry, thus prompting epimerization with NaOCH_3_. The epimerized product gave the desired cycloadduct, which was then deprotected and oxidized to yield **25**. Further steps led to punctatins A and D.

**Scheme 5:**
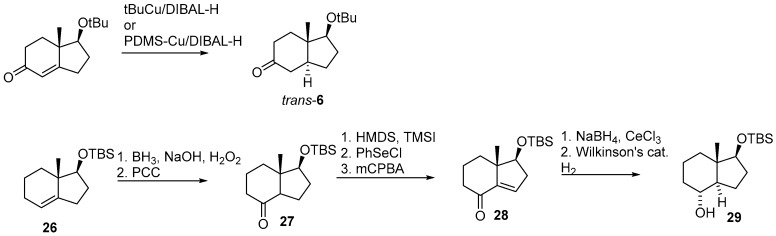
Reduction of **1** and **2** to afford *trans*-hydrindane scaffolds.

Until this point, Hajos and Parrish’s work for generating the *trans*-hydrindane was used as a standard methodology, with little further development on the reduction of the enone moiety. In 1988, the use of DIBAL-H with *tert*-butylcopper was found to selectively and in good yield reduce the enone to the *trans*-hydrindane (see Scheme 5) [21]. In 2001, this was investigated again using silyl copper systems, finding that phenyldimethylsilylcopper is more efficient at producing the *trans*-hydrindane [22]. A separate method for introducing the *trans*-ring juncture came from **26** [23]. Then, transformation of **26** through hydroboration/oxidation to ketone **27**, then trapping of the HMDS generated enolate with TMS-I allowed for treatment of the enol ether with PhSeCl, and subsequent oxidation by mCPBA afforded enone **28**. Luche reduction with NaBH_4_/CeCl_3_ comes from the β-face of the hydrindane, and the resulting alcohol acts as a directing group for reduction with Wilkinson’s catalyst to give *trans*-hydrindane **29**. The authors note here that Crabtree’s catalyst for this system leads to the *cis* isomer [23].

**Scheme 6:**
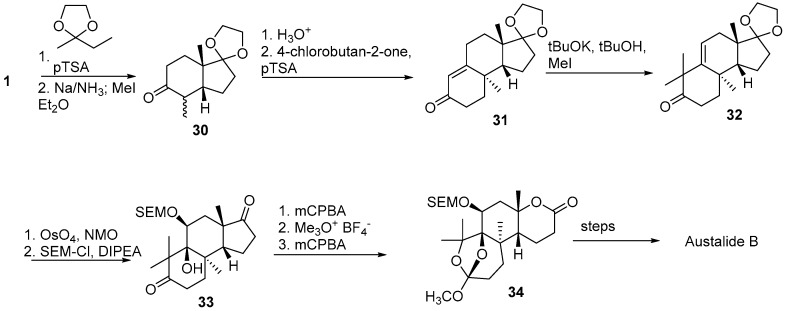
Synthesis of austalide B core from **1**.

Hydrindane **1** again found use in the total synthesis of austalides, meroterpenoids from *Aspergillus ustus* (see Scheme 6) [24,25]. In this work, protection of **1** followed by Birch reduction in the presence of MeI gave **30**, which was then treated with acid and 4-chlorobutan-2-one to give **31** as a single diastereomer. Polymethylation under the action of MeI in *tert*-BuOK/*tert*-BuOH afforded the dimethylated system **32**. Dihydroxylation with OsO_4_/NMO, and protection with SEM-Cl gave **33**. Baeyer–Villiger oxidation with mCPBA was found to occur in the A ring first, and allowed for trapping of the *ortho*-ester with Me_3_O^+^ BF_4_^−^ to afford **34**, after a second Baeyer–Villiger. Further steps finalized the synthesis of austalide B.

**Scheme 7:**
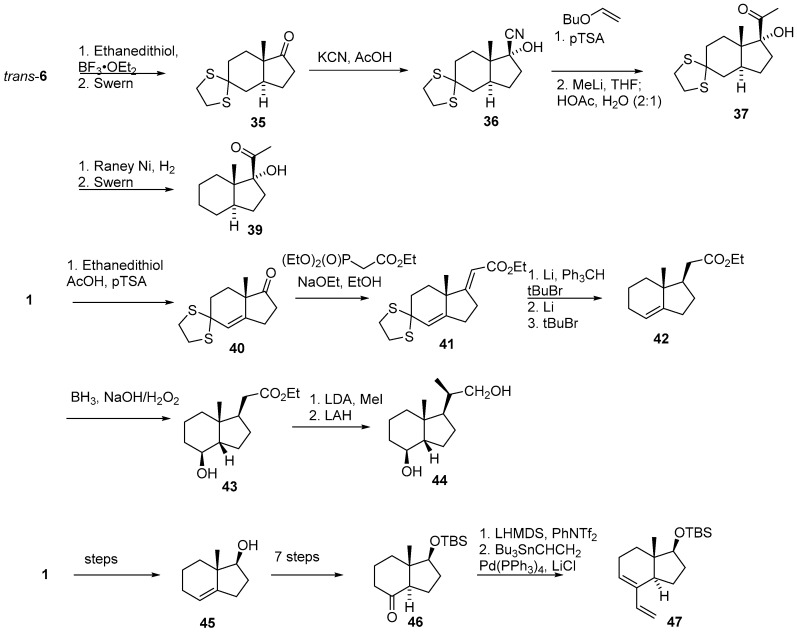
Derivatizations of **1** and *trans*-**6** for SAR studies.

Terpene and terpenoid derivatives are well known to hold significant bioactivity, thus it should be no surprise that the bicyclo[4.3.0]nonane scaffold should also possess some selectivity relevant for medical applications. Indeed, this is true, and more surprisingly, selectivity in some cases is driven by substitution of the hydrindane [10,11,26,27]. Progesterone analogues were discovered to inhibit angiogenesis in conjunction with heparin (see Scheme 7) [11]. Here, it was found that the C-17 a-hydroxy functionality was important for the anti-angiogenic activity. Even when using the hydrindane moiety alone, it was found that the anti-angiogenic activity was retained. In this work, *trans*-**6** was protected as dithiane, and oxidized via Swern’s conditions to give **35**. Cyanohydrin formation led to attack on the β face, giving **36**. Transformation of the cyanide into methyl ketone **37**, followed by complete removal of the dithiane with Raney nickel and reoxidation led to structural derivatives of the progesterone C/D rings. Vitamin D_3_ analogues have also been utilized in structure-activity relationship (SAR) studies [28]. Hydrindane **1** was protected as the dithiane, **40**, then olefinated through Horner–Wadsworth–Emmons conditions to give α,β-unsaturated ester **41**. Tandem reduction steps led to the removal of the dithiane and conjugated alkene, leaving the isolated alkene untouched (**42**). Hydroboration/oxidation led to β-hydroxy **43**, and further transformed into the Inhoffen–Lythgoe diol, **44**. Later, formulation of an ethenyl substitution for application to (+)-estradiol was found to be higher yielding than the previous sequence [29]. Following previously published conditions, this work took **1** on to form **45**, and further into **46**. Enolate formation with LHMDS and trapping with *N*-phenyltriflimide led to the enol triflate precursor for Stille coupling with vinyl tributylstannane, generating **47**. Isolated **47** was proposed as a short sequence leading to Diels-Alder diene precursors primed for formation of triterpene and triterpenoid B rings.

**Scheme 8:**
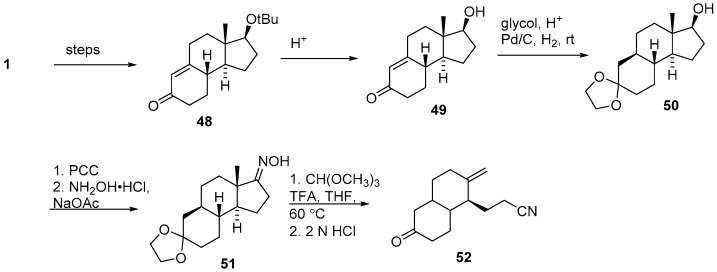
Advanced intermediate for solanopyranones.

Solanopyrones, potent inhibitors of DNA polymerase β and λ, have been synthesized utilizing a Beckmann fragmentation of the hydrindane core to reveal the proper stereochemistry necessary for the pyranone moiety (see Scheme 8) [30]. Under known conditions, **1** was transformed into **48**, and deprotected to reveal the hydroxyl **49**. Protection with concomitant reduction of the α,β-unsaturated ketone led to **50**, which was oxidized and transformed into oxime **51**. Beckmann fragmentation followed by deprotection of the dioxolane led to **52** as the desired intermediate.

**Scheme 9:**
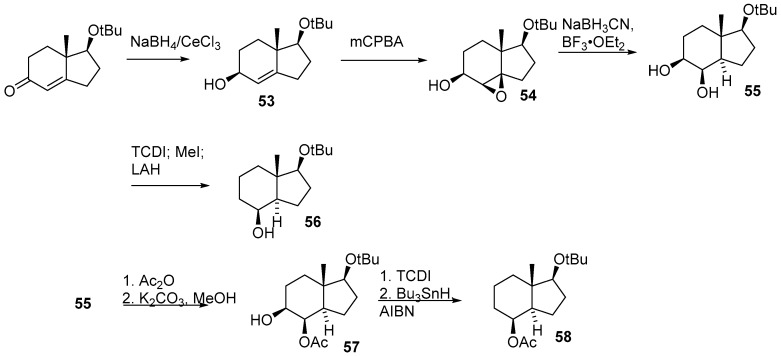
Rapid entry to calcitriol (vitamin D_3_) hydrindane.

As another approach to the vitamin D_3_ hydrindane intermediate, an approach utilizing the α,β-unsaturated ketone as a directing group was explored in two accounts (see Scheme 9) [31,32]. Luche reduction of the ketone, **5**, with NaBH_4_/CeCl_3_ afforded the allylic alcohol with good diastereoselectivity, giving **53** [31]. Upon epoxidation with mCPBA (**54**), and reduction with Hutchin’s conditions of NaBH_3_CN–BF_3_·OEt_2_, the *trans*-hydrindanediol **55** was produced. Further reaction with thionocarbonyl diimidazole (TCDI), methyl iodide, and reduction by LAH allowed for selective reduction of the less hindered side of the diol to afford the trans-hydrindanol **56**. In a subsequent account [32], Wicha showed that acylation with Ac_2_O of **55** along with selective removal of the less hindered ester by K_2_CO_3_ gave **57**, which was treated with TCDI and reduced with Bu_3_SnH/AIBN to afford **58** in six steps with an overall yield of 63% from **5**.

**Scheme 10:**
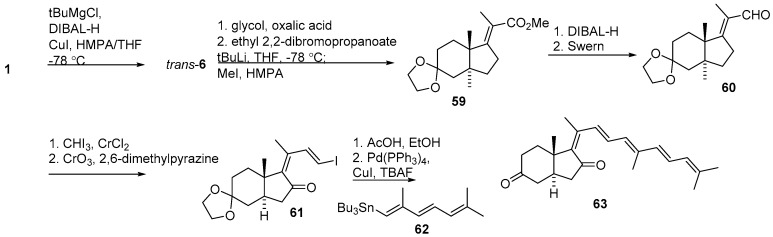
Globostellatic acid SAR derivative synthesis.

**1** found use in the synthesis of the methyl ester of globostellatic acid X, a selective inhibitor of human endothelial umbilical vein cell proliferation (see Scheme 10) [33]. Treatment of **1** with *tert*-butylcopper and DIBAL-H in HMPA/THF at −78 °C allowed for the generation of *trans*-**6**, which was protected as the dioxolane and then olefinated with ethyl 2,2-dibromopropanoate/*tert*-butyllithium. Quenching the reaction mixture with MeI afforded **59**. Reduction/oxidation using DIBAL-H followed by Swern’s conditions for oxidation provided α,β-unsaturated aldehyde **60**, which was transformed into the alkenyl iodide **61** through the action of iodoform and chromium (II) chloride followed by oxidation with CrO_3_. Acidic deprotection of the dioxolane, and Stille coupling with **62** led to globostellatic acid derivative **63** which were used for SAR studies.

**Scheme 11:**
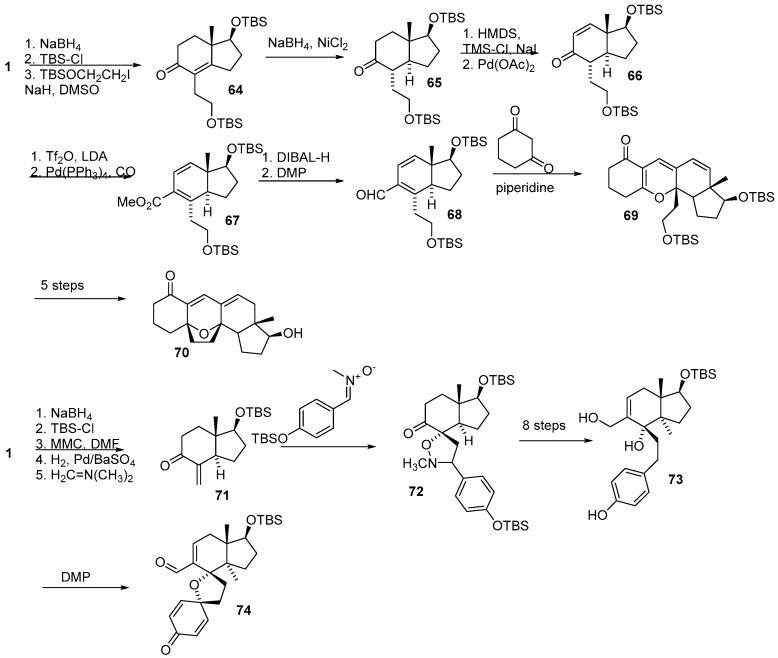
Hirama and Sorenson routes to cortistatin framework.

Cortistatins, belonging to the triterpene alkaloids class, pose a particular synthetic challenge. Recent routes have been attempted, with two being successful (see Scheme 11) [34,35,36]. In 2008, Hirama proposed using **1** to undergo a Knovengel condensation/electrocyclic reaction/radical cyclization strategy to afford cortistatin A- the most potent cortistatin [34]. Hydrindane **1** was transformed into **64** by NaBH_4_ reduction, protection of the alcohol with TBS-Cl, and alkylation. Reduction of **64** with NaBH_4_/NiCl_2_ allowed for production of the *trans*-hydrindane **65**, and further treated with HMDS in the presence of TMS-Cl and NaI to afford the trimethylsilyl enol which was oxidized to α,β-unsaturated ketone **66**. Triflation and palladium catalyzed carbonylation in methanol afforded ester **67**, which underwent reduction with DIBAL-H and oxidation with DMP to afford aldehyde **68**. Knovenagel condensation of **68** with 1,3-cyclohexanedione afforded **69**, which was able to be turned into key intermediate **70** for cortistatins in relatively few steps. Sorenson also put forth an approach to the cortistatin framework utilizing **1** and envisioned phenolic oxidation to form the furan bicyclic fusion [35]. Hydrindane **1** was transformed into the TBS ether, **71**, and reacted with methyl magnesium carbonate. A series of hydrogenation with Pd/BaSO_4_ yielded the *trans*-hydrindane, which was exposed to Eschenmoser’s salt to afford the exocyclic alkene with loss of CO_2_. Under thermal conditions, 1,3-dipolar cycloaddition with the nitrone led to the formation of 1,2-isooxazolidine, **72**. Triflation of the ketone followed by palladium catalyzed carbonylation led to a methyl ester. A short sequence led to pendant alcohol system **73**, which was oxidized to allow for formation of the spiro-tetrahydrofuran moiety through phenolic oxidation. Nicolau also proposed a route to the eastern portion of cortistatin A, using **1** [36].

**Scheme 12:**
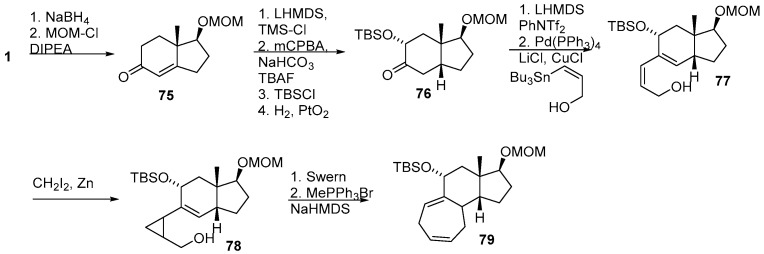
Sarpong’s approach to cyathane and cyanthiwigin scaffolds.

Cyathane and cyanthiwigin diterpenoids have also been synthesized via **1** through a parallel kinetic resolution strategy (see Scheme 12) [37]. Hydrindane **1** was transformed into the MOM ether **75** through reduction and protection. Further transformation by Rubottom oxidation conditions and protection of the resulting alcohol with TBS-Cl, allowed for hydrogenation with Adam’s catalyst to give the formation of **76**. Triflation and Stille coupling gave the dienol **77**, which went exposed to Simmons–Smith cyclopropanation yielded the vinylcyclopropane **78**. Swern oxidation and Wittig olefination led to the cycloheptadiene scaffold, **79**, via a Cope rearrangement.

**Scheme 13:**
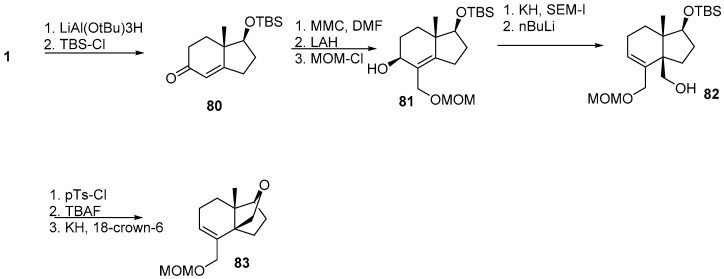
Synthesis of advanced intermediate **83** for xenibellol total synthesis.

Xenibellol, another diterpenoid found to be cytotoxic to P-388 cells, has been synthesized from **1** (see Scheme 13) [38]. Reduction and protection led to TBS ether **80**. Treatment with MMC and LAH reduction led to a diol, which was selectively protected as the MOM ether to generate **81**. Formation of the SEM ether allowed for 2,3-Wittig rearrangement with n-BuLi to give **82**. Tosylation, followed by TBAF deprotection and etherification led to the advanced intermediate **83**.

**Scheme 14:**
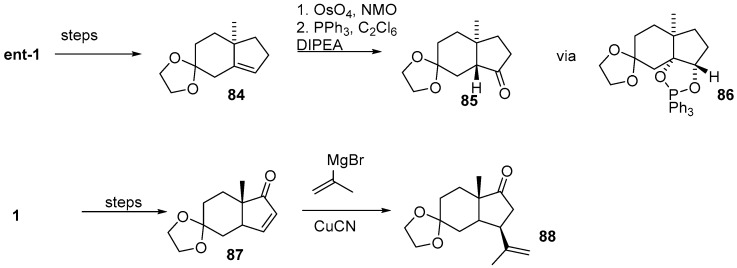
Modifications of **1** for natural product synthesis.

In the last decade, other modifications to hydridane cores have been explored. In the synthesis of dictyoxetane’s hydrindane, ent-**1** was transformed into **84**, then dihydroxylated with OsO_4_/NMO to give a diol (see Scheme 14) [39]. Here the authors found a unique method for formation of the *trans*-hydrindane core by treating the diol with PPh_3_, C_2_Cl_6_, and Hunig’s base in acetonitrile to afford **85**. Putatively, the transient phosphinite ether, **86**, undergoes pinacol rearrangement to yield the desired hydrindane. Similarly, a unified approach to *trans*-hydrindanes common to an array of sesterterpenoids was put forth by Trauner [40]. In this work, **1** was transformed into the *tert*-butyl ether, **5**, then further into the *trans*-hydrindane, *trans*-**6**, along the path outlined by Hajos and Parrish. Protection of the ketone, and removal of the *tert*-butyl ether followed by oxidation allowed for a ketone to be established for subsequent installation of the α,β-unsaturated system, **87**. Cuprate addition led to formation of **88**, which holds the appropriate substitution for further synthesis of natural products. This unified approach was later shown in the synthesis of (+)-nitidasin [41].

**Scheme 15:**
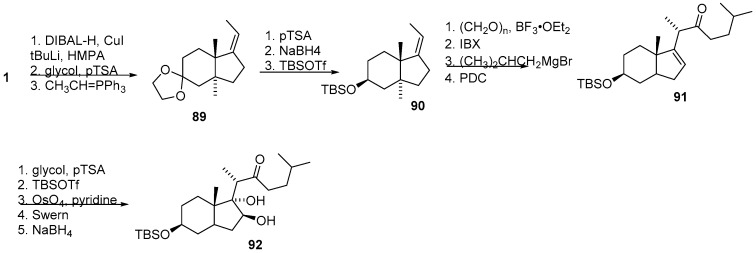
Synthesis of OSW-1 CD rings.

OSW-1, a steroidal glycoside, has been found to possess potent anti-cancer effects (see Scheme 15) [42]. The des-AB aglycone have been also found to possess potent inhibitory effects. Hydrindane **1** was reduced with DIBAL/*tert*-butylcopper, then protected as the dioxolane, and olefinated to give **89**. Deprotection, followed by reduction and protection led to the TBS ether, **90**. A methanol moiety was installed via paraformaldehyde/boron trifluoride, then oxidized to reveal the aldehyde. Grignard addition with isobutylmagnesium bromide and reoxidation generated intermediate **91**. Under acidic deprotection, removal of the TBS group was found along with production of the dioxolane, which was protected with TBS-OTf to form the desired product. A sequence of dihydroxylation, oxidation, and reduction led to the formation of the desired *trans*-hydrindane core, **92**, for OSW-1.

**Scheme 16:**
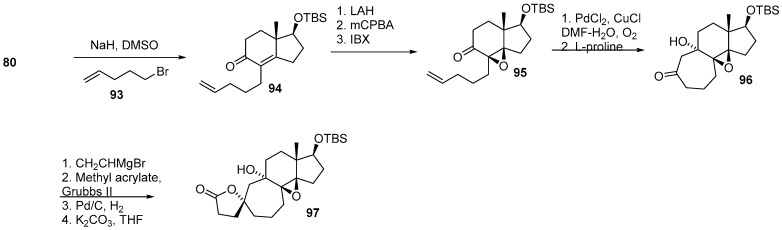
Synthesis of shiartane scaffold from **80**.

**1** has also been transformed into the core of shiartane type 1 nortriterpenoids (see Scheme 16) [43]. Utilizing the known transformation to **80**, sodium hydride and bromide **93** were used to generate scaffold **94**. LAH reduction followed by hydroxy directed epoxidation with mCPBA led to a chiral epoxide, which was further oxidized with IBX to give **95**. Wacker–Tsuji oxidation gave a diketone, which allowed for cyclization with l-proline to afford **96**. Grignard addition of vinylmagnesium bromide followed by cross metathesis with methyl acrylate allowed for elaboration of the western portion of the shiartane scaffold. Further reduction and esterification completed the western portion, giving **97**.

The work done with **1** has provided a great benefit to total synthesis, but is not without its limitations. As shown, the *trans*-hydridane, *trans*-**6**, is complicated by the open β-face of **5**, giving a more facile reduction to lead to the *cis*-isomer. Above, the published results have shown that reactions are available to circumvent this preferred pathway, while allowing for further elaboration of the core structures necessary for the desired target. However, the length of these sequences can be deleterious to yield, and thus other methods have been explored for more rapid, efficient, and higher yielding syntheses. These reactions are presented below.

### 2.2. Cyclization Strategies to Hydrindane Cores

As functionalization of **1** often requires lengthy steps with protection and deprotection steps in order to achieve selective reaction, as well as having problems directly accessing a *trans*-hydrindane scaffold. A common strategy for negating the deleterious effect of the fused 6,5-bicyclic structure is to directly form it in a single step through cycloaddition methodology; Diels-Alder reactions, metathesis reactions, and Michael additions.

**Scheme 17:**
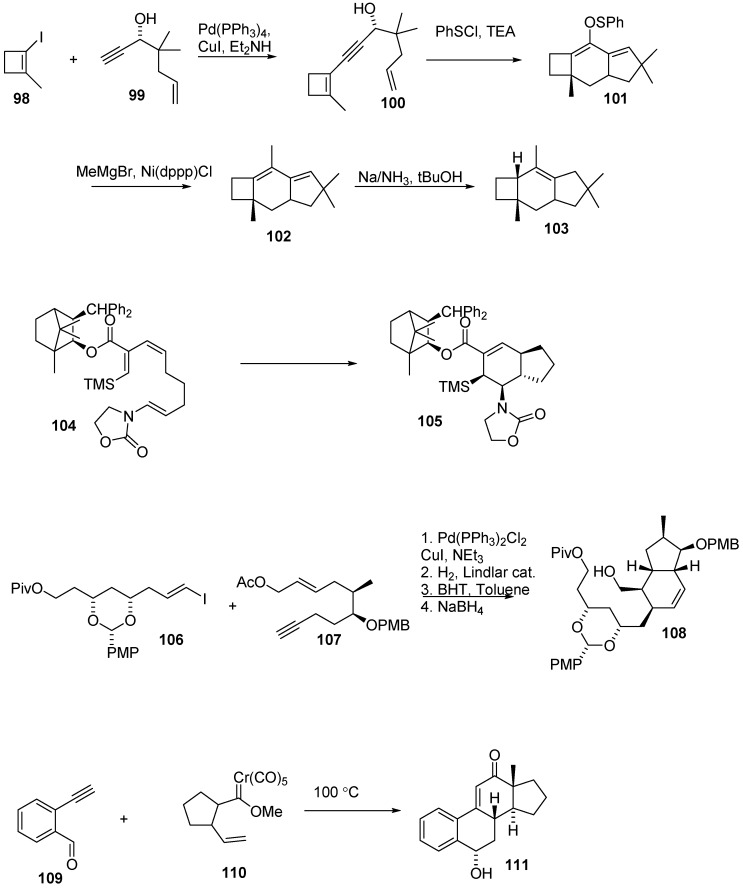
Cycloaddition strategy to hydrindane scaffolds.

The Diels-Alder reaction, which has been reviewed extensively [44,45,46,47,48], provides a systematic way of introducing contiguous stereocenters with relative ease. One of the main advantages of the Diels-Alder cycloaddition is the overall understanding of its reactivity, selectivity, and methods for chiral induction. Using vinylallene sulfoxides in the synthesis of sterpurene [49,50], chiral alcohol **99** was coupled to cyclobutenyl iodide **98**, then transformed into intermediate **101** through the action of PhSCl (see Scheme 17). This intermediate underwent a facile cyclization to afford the desired core, and after treatment with MeMgBr/Ni(dppp)Cl and Na/NH_3_, sterpurene (**103**) was isolated. A second example of the intramolecular Diels-Alder is the use of chiral 1,3-butadiene-2-carboxylates bearing a pendant chiral modifier [51]. Here, when using oxazolidinone **104**, **105** was obtained in good yield with high diastereoselectivity (ca. 17:1). Likewise, advanced intermediate **108** was proposed from the combination of iodide **106** and alkyne **107** after coupling, Lindlar reduction, and cycloaddition [52]. Of note, the chromium carbene **110** can also be used to simultaneously form B and C rings in steroidal systems when treated with **109**. [53] Similar methodology was applied to the synthesis of galliellalactone, which inhibits IL-6 signaling implicated in oncogenic pathways [54]. 

**Scheme 18:**
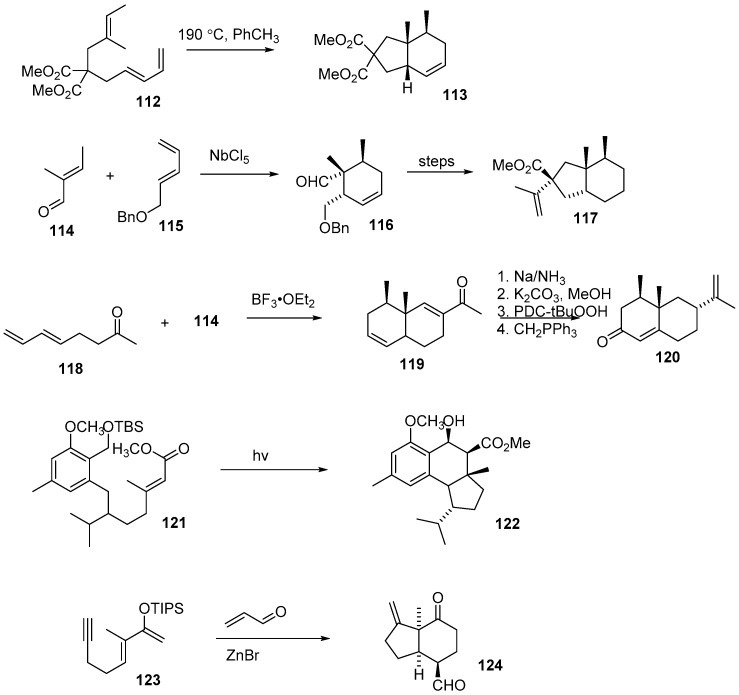
Diels-Alder cascades for hydrindane synthesis.

A benefit to the Diels-Alder is that it requires so little to effect cyclization, and can be used in combination with other reactions (see Scheme 18). An approach to the bakkane skeleton through successive alkylations of dimethylmalonate led to Diels-Alder precursor **112**, which provided a *cis*-hydrindane **113** for further elaboration [55,56]. More recent work has expounded upon this route to utilize **118** and α,β-unsaturated aldehydes to provide adducts similar in nature to **114**, and has been applied to the synthesis of nootakone [57]. In addition to these ideas, a diene can be generated through photoenolization of benzaldehyde derivatives [58]. This idea has been applied to the hamigeran series, where an advanced intermediate **121** was exposed to light to catalyze the photoenolization and allowed to undergo resulting cycloaddition to **122**. A Diels-Alder/carbocyclization has also been shown to give *cis*-hydrindane products through the action of zinc salts [59]. Here, dieneyne **123** reacts with acrolein to undergo a primary Diels-Alder, and spontaneously cyclizes to hydrindane **124**. Across a range of substrates, up to 96% yield was obtained with excellent diastereoselectivities (ca. 17:1).

**Scheme 19:**
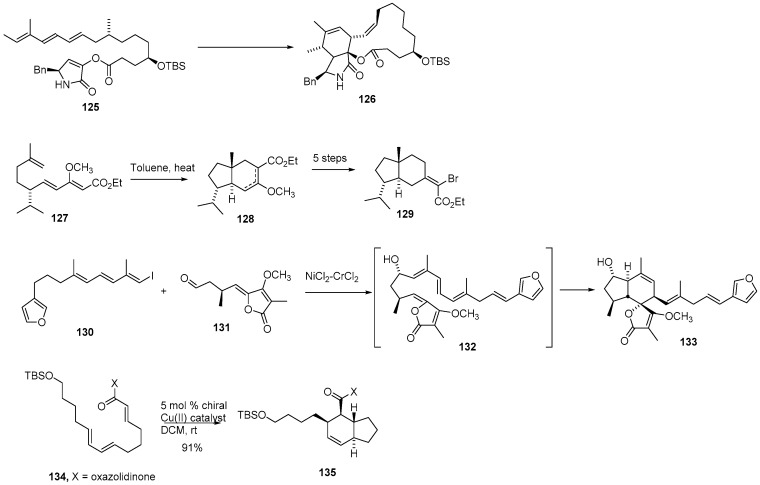
Utilization of intramolecular Diels-Alder reactions in natural product synthesis.

The use of the intramolecular Diels-Alder in natural product synthesis has been found valuable in the synthesis of polycyclic structures. Stork showed that naturally occurring cytochalasins were amenable to a targeted intramolecular Diels-Alder (see Scheme 19, **125** to **126**) [60]. In 1989, Hudlicky approached the total synthesis of (−)-retigeranic acid with similar methodology [61]. In Hudlicky’s synthesis, **127** was converted through the intramolecular Diels-Alder to a set of regioisomers, **128**. Five steps resolved the regioisomeric mixture into the desired natural product, **129**. Uenishi later followed by generating the requisite [4+2] diene–dienophile pair, **130** and **131**, through nickel–chromium catalyzed bond formation to give intermediate **132** that spontaneously cyclized to **133** for the synthesis of ircinianin and wistarin [62]. Evans and Johnson in the same year utilized a chiral copper reagent to effect an efficient intramolecular Diels-Alder cycloaddition to afford *trans*-hydrindane **135** in 91% from **134** for use in the synthesis of (−)-isopulo’upone [63].

**Scheme 20:**
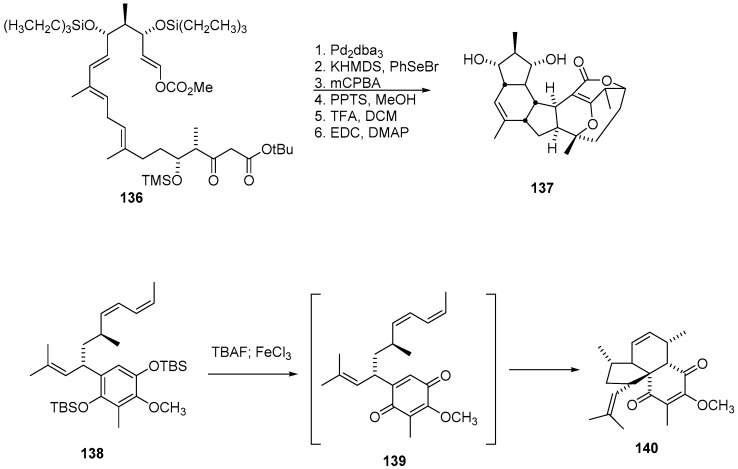
Synthesis of (+)-FR182877 and elisabethinin A.

Sorenson in 2002 utilized a set of tandem intramolecular Diels-Alder reactions for (+)-FR182877 (see Scheme 20) [64]. In this work, the tetrene **136** was synthesized, then went through a macrocyclization reaction under the action of Pd_2_dba_3_. A subsequent sequence of five reactions following the proposed biosynthetic route led to completion of the molecule. Heckrodt in 2003 showed a similar intramolecular Diels-Alder inspired by biomimetic conditions of elisabethinin A [65]. Here, the authors built aryl triene **138**, and the sequence of deprotection via TBAF and phenolic oxidation of FeCl_3_ allowed for generation of an intermediate benzoquinone that underwent spontaneous trapping with the pendant diene to afford the final product in 91%.

**Scheme 21:**
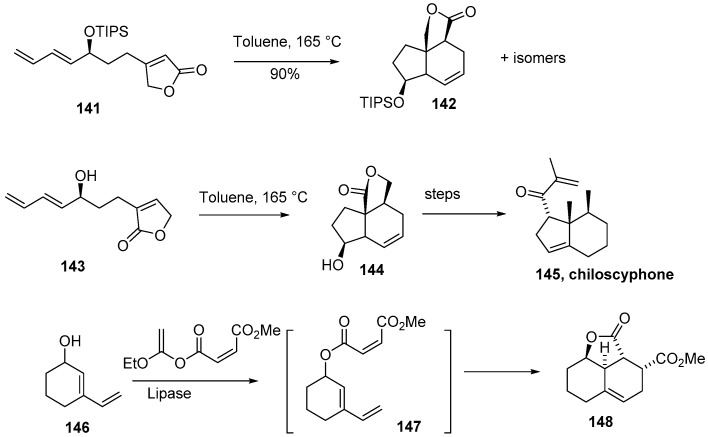
Examples of intramolecular Diels-Alder reactions leading to tricyclic cores.

Shiina and Nishiyama applied the intramolecular Diels-Alder to deriving tricyclic derivatives of the *trans*-hydrindane core (see Scheme 21) [66]. Tethered diene **141** under sealed tube conditions in toluene led to the tricyclic core **142**, which was later utilized as derivative **143** in the racemic synthesis of chiloscyphone (**145**) and isochiloscyphone [67]. Akai et al. showed that lipase resolution allowed for tandem dynamic kinetic resolution of racemic alcohols, **146**. Subsequent trapping with an intramolecular Diels-Alder reaction led to the formation of complex tricycles with four contiguous stereocenters set with high levels of enantioinduction [68].

**Scheme 22:**
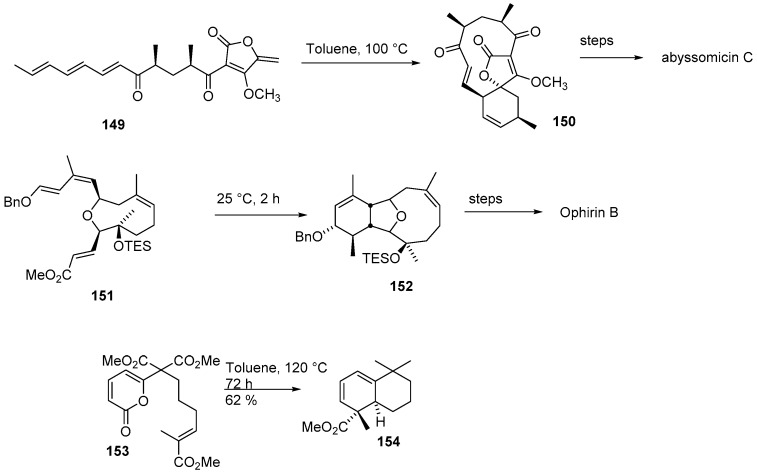
Intramolecular Diels-Alders potentially applicable to hydrindane synthesis.

Other notable examples of intramolecular Diels-Alder reactions that are able to form hydrindane cores have appeared in the literature, but have yet to show direct use (see Scheme 22). Sorenson utilized polyene **149** in a route toward the synthesis of abyssomicin C, ultimately forming the spiro[6.5] ring juncture through an intramolecular Diels-Alder reaction [69]. Crimmins and Brown utilized an approach with **151** as an intermediate to synthesize ophirin B [70]. Lastly, Stoltz applied an intramolecular Diels-Alder utilizing pyrone **153** as a key intermediate in the progress to basiliolides and transtaganolides [71].

**Scheme 23:**
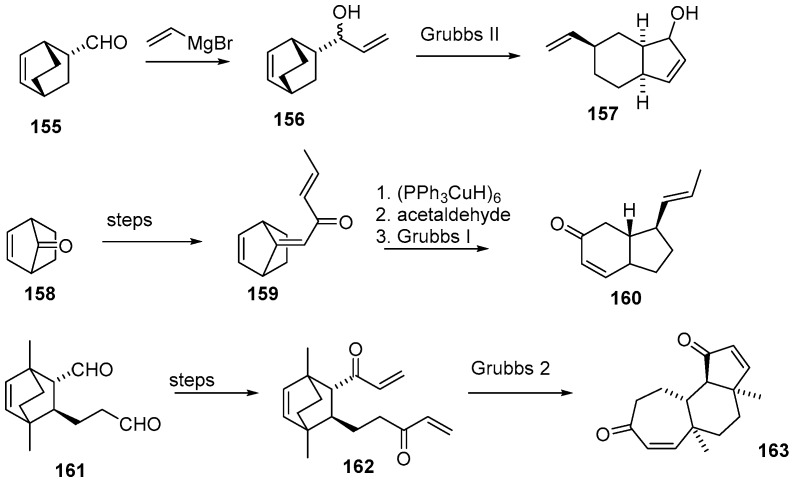
Metathesis strategies for hydrindane formation.

Metathesis as a cyclization tactic has become a notable method for ring forming reactions. The use of Grubbs or Grubbs–Hoveyda catalysts has often been the choice of catalyst used for these reactions, and their involvement in the synthesis of hydrindanes has become documented (see Scheme 23) [72,73]. Bicyclo[2.2.2]octenes are useful substrates for these reactions because they are readily synthesized from Diels-Alder cycloadditions, and can be utilized to give hydrindane scaffolds with a variety of functionalizations. Derivative **155** was derivatized with vinylmagnesium bromide and then exposed to Grubbs second generation catalyst to yield hydrindane **157** in 93% [74]. Later explorations led to the derivative **159** to undergo ROM/RCM cleavage to give alkenyl substituted hydrindane **160** [75]. Dialdehyde **161** was transformed into **162** and exposed to Grubbs second generation to give [7.6.5]tricycle **163** [76]. Enyne metathesis coupled to Diels-Alder cycloaddition has also been applied to this type of methodology [77]. Further, a sequential ring closing metathesis followed by Heck coupling was shown in 2010, giving a *trans*-hydrindane core for the C/D ring juncture [78]. This methodology has been applied to the synthesis of norrisolide [79].

**Scheme 24:**
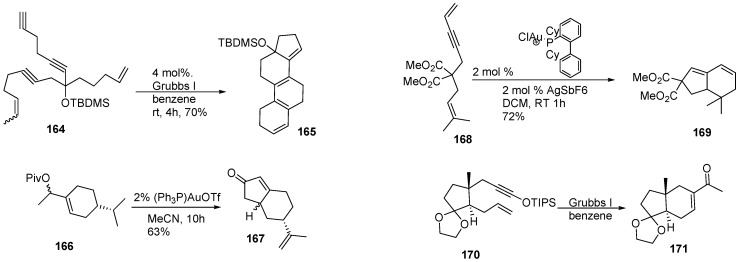
Enyne metathesis reactions leading to hydrindane cores.

Enyne metathesis has found an increasing interest in the synthesis of natural products (see Scheme 24) [80,81,82,83,84]. It has found significant use in the synthesis of larger ring systems [85,86,87,88,89] and hetereocyclic systems [90,91,92,93,94,95], but only occasionally in the synthesis of hydrindanes [96,97,98,99]. Palladium, platinum, gold, and ruthenium have been found to be the catalysts of choice for this class of reactions. Grubbs has shown that his catalyst was able to efficiently form triterpenoid system **165** from **164** [100]. Cyclohexenyl system **166** forms hydrindone **167** when exposed to triphenylphosphinegold triflate in modest yield [98]. Dienyne **168** has been shown on three accounts to form substituted hydrindadiene **169** in good yields utilizing gold catalysts [86,87,91]. Cyclopentane system **170** under the action of Grubbs I allowed for hydrindane **171** to be formed [97].

**Scheme 25:**
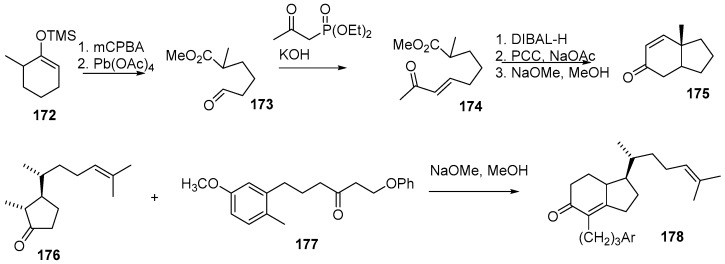
Michael additions for hydrindane scaffold.

Cycloaddition and metathesis reactions give rapid entry into the hydrindane core, but are not the only pathway available to their synthesis. Along with Michael additions, as in the synthesis of **1**, Morita–Baylis–Hillman reactions and radical reactions can be used to form the bicyclic core (see Scheme 25). Their use, however, does add to a sequence’s number of steps in order to judiciously choose substrates that provide the desired stereochemical outcome. Oxidation of the trimethylsilyl ether **172** with mCPBA affords the Rubottom product, **173** [101]. Further oxidation by lead tetraacetate and Horner–Wadsworth–Emmons olefination led to ketoester **174**. A reduction-oxidation sequence to garner the requisite dialdehyde ultimately provided *trans*-hydrindene **175** after treatment with sodium methoxide in methanol. Similarly, utilization of cyclopentanone **176** with ketone **177** led to formation of hydrindene **178** in 78% [102]. Other examples have appeared in the literature [103,104,105,106].

**Scheme 26:**
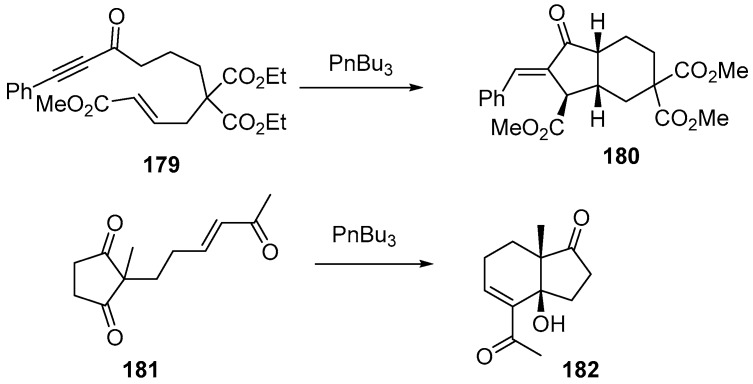
Morita–Baylis–Hillman reactions leading to hydrindanes.

In a similar vein as the Michael additions, the Morita–Bayliss–Hillman under phosphine or transition metal catalysis has been shown to provide similar results to Michael addition strategies (see Scheme 26). Using enyne **179** with tri-n-butylphosphine, *cis*-hydrindane **180** was generated [107]. Likewise, the *cis*-hydrindane **182** was found to be formed from a full equivalent of tri-*n*-butylphosphine with triketone **181** [108]. Lastly, the titanium variant of the Morita–Bayliss–Hillman using **181** gives **182**, stemming from the thermodynamic Z-titanium enolate that forms as an intermediate [109].

## 3. Summary and Outlook

Here, we have presented methodology that has been used for the production of the hydrindane core. The difficulty associated in the generation of this scaffold lies not in the difficulty of the reactions leading to the core, but rather lies in the thermodynamic nature of the *cis*-hydrindane core’s stability. This has been alleviated through judicious choice of substrates, either through derivatization to change thermodynamic stability or through development of scaffolds prone to react in the desired manner. With the propensity of natural products bearing this moiety, it becomes necessary to discuss the synthesis of the hydrindane because of its inherent bioactivity (vide supra) and the number of stereocenters distributed over the scaffold. Thus, the hydrindane motif serves a dual purpose: (1) a synthetic challenge; and (2) a lead compound for drug development. As shown in this review, there has been a large volume of work done representing a broad range of reactions and elaborations of the hydrindane nucleus that greatly benefit natural product synthesis, as well as medicinal chemistry.
